# On the Feasibility of Using an Ear-EEG to Develop an Endogenous Brain-Computer Interface

**DOI:** 10.3390/s18092856

**Published:** 2018-08-29

**Authors:** Soo-In Choi, Chang-Hee Han, Ga-Young Choi, Jaeyoung Shin, Kwang Soup Song, Chang-Hwan Im, Han-Jeong Hwang

**Affiliations:** 1 Department of Medical IT Convergence Engineering, Kumoh National Institute of Technology, Gumi 39177, Korea; sooin1118@naver.com (S.-I.C.); cgy326@naver.com (G.-Y.C.); kssong10@kumoh.ac.kr (K.S.S.); 2 Berlin Institute of Technology, Machine Learning Group, Marchstrasse 23, 10587 Berlin, Germany; zeros8706@naver.com; 3 Department of Biomedical Engineering, Hanyang University, Seoul 04763, Korea; naraeshigo@gmail.com (J.S.); ich@hanyang.ac.kr (C.-H.I.)

**Keywords:** ear-EEG, brain-computer interface (BCI), electroencephalography (EEG), mental arithmetic, endogenous BCI

## Abstract

Brain-computer interface (BCI) studies based on electroencephalography (EEG) measured around the ears (ear-EEGs) have mostly used exogenous paradigms involving brain activity evoked by external stimuli. The objective of this study is to investigate the feasibility of ear-EEGs for development of an endogenous BCI system that uses self-modulated brain activity. We performed preliminary and main experiments where EEGs were measured on the scalp and behind the ears to check the reliability of ear-EEGs as compared to scalp-EEGs. In the preliminary and main experiments, subjects performed eyes-open and eyes-closed tasks, and they performed mental arithmetic (MA) and light cognitive (LC) tasks, respectively. For data analysis, the brain area was divided into four regions of interest (ROIs) (i.e., frontal, central, occipital, and ear area). The preliminary experiment showed that the degree of alpha activity increase of the ear area with eyes closed is comparable to those of other ROIs (occipital > ear > central > frontal). In the main experiment, similar event-related (de)synchronization (ERD/ERS) patterns were observed between the four ROIs during MA and LC, and all ROIs showed the mean classification accuracies above 70% required for effective binary communication (MA vs. LC) (occipital = ear = central = frontal). From the results, we demonstrated that ear-EEG can be used to develop an endogenous BCI system based on cognitive tasks without external stimuli, which allows the usability of ear-EEGs to be extended.

## 1. Introduction

Neurological diseases, such as amyotrophic lateral sclerosis, brainstem strokes, and spinal cord injuries, could lead to locked-in syndrome (LIS), which makes it impossible for LIS patients to have full voluntary muscle control [1]. Recently, interest in brain-computer interfaces (BCIs) has increased because they can replace the function of LIS patients’ impaired bodies.

BCI allows LIS patients to communicate with the external environment using only brain signals without any voluntary movements [2]. In other words, BCI systems can provide alternative communication channels for LIS patients [3]. BCIs can be realized using various neuroimaging modalities, such as electrocorticography (ECoG) [4,5], electroencephalography (EEG) [6,7], magnetoencephalography (MEG) [8,9], functional near-infrared spectroscopy (fNIRS) [10,11], and functional magnetic resonance imaging (fMRI) [12,13]. An invasive signal recording technique, such as ECoG, requires a surgical operation to place recording electrodes on the cortex. In contrast, non-invasive techniques, such as EEG, MEG, fNIRS, and fMRI, can provide safe recording of brain activity without surgery. Thus, the majority of BCI systems have been implemented based on non-invasive neuroimaging modalities [14]. Among the noninvasive modalities, EEG has been widely used to develop BCIs because it has high portability, reasonable cost, and high temporal resolution compared to other noninvasive recording modalities [14].

Conventional EEG-based BCI systems have used brain signals measured using scalp electrodes with conductive gels for accurate measurement of EEGs. However, the conventional measurement system is relatively bulky because it consists of several components, such as an external amplifier, cap, and electrode, which limits the value of BCI applications in terms of practical use. Recently, to overcome the limitation of conventional EEG-based BCI systems, EEGs measured by electrodes attached around the ears, called ear-EEGs, have been proposed as an alternative to the classical scalp-EEG [15,16]. It has been proven that ear-EEG can be set-up within several minutes, and used for successive days [17,18,19]. Most importantly, the feasibility of ear-EEG-based BCI systems has been demonstrated even though their performance is still lower than that of conventional EEG-based systems in general [18,20].

There are two approaches to develop EEG-based BCI systems according to whether external stimuli are used or not [21]. The first is based on an exogenous BCI paradigm that uses external stimuli to induce specific brain patterns, such as auditory steady-state responses (ASSRs) [22,23], event-related potentials (ERPs) [17,24,25,26,27,28], and steady-state visual evoked potentials (SSVEPs) [29]. The other approach is based on an endogenous BCI paradigm that uses self-modulated EEG patterns without external stimuli [30,31,32,33], such as sensorimotor rhythms [34] and slow cortical potential (SCP) [35]. Irrespective of if an exogenous or endogenous BCI system is developed, the most important factor to consider is practicality for clinical use and daily applications. Most previous BCI studies based on ear-EEG have used exogenous paradigms with external stimuli because of relatively high signal-to-noise ratio (SNR) compared to the SNR of endogenous paradigms. However, repetitive presentation of external stimuli used in exogenous BCI paradigms can cause user fatigue [36,37,38], and additional hardware is required to provide visual, auditory, or tactile external stimuli. The mentioned disadvantages would make it difficult to use an exogenous BCI system over a long period in daily life. Therefore, an endogenous BCI can be an alternative to the exogenous BCI in terms of practical use, but the feasibility of using ear-EEG to develop an endogenous BCI system has rarely been evaluated in previous BCI studies. 

The objective of this study was to demonstrate the feasibility of ear-EEG for the development of an endogenous BCI system. To this end, EEGs were measured around the ears as well as on the scalp while subjects performed mental arithmetic (MA) and light cognitive (LC) tasks (imagining vocalization of English letters from A to Z). We compared event-related (de)synchronization (ERD/ERS) patterns induced by the MA and LC tasks between scalp-EEG and ear-EEG, and the classification accuracies of the MA and LC tasks to test the feasibility of an ear-EEG-based endogenous BCI system. As a result, similar ERD/ERS patterns were obtained between scalp- and ear-EEG during both MA and LC tasks. Also, the mean classification accuracy of ear-EEG was comparable to those of other scalp areas, and it was above 70% required for effective binary communication, demonstrating that the feasibility of using ear-EEG for the development of an endogenous BCI system.

## 2. Materials and Methods

### 2.1. Subjects

Eighteen healthy individuals were recruited for this study (21–31 years of age; mean 24.5 ± 2.67 years, 10 males and eight females). They reported no history of neurological or psychiatric conditions. All subjects were informed about the experimental procedure, and written consent was obtained from each subject before the experiment. All subjects received financial reimbursement after the experiment. The experimental protocol of this study was approved by the Institutional Review Board (IRB) of Kumoh National Institute of Technology (No. 6250). EEG data analysis was conducted for fifteen subjects, excluding three subjects who reported excessive fatigue during the experiment, and drinking alcohol in the previous day, which could affect experimental results.

### 2.2. EEG Measurement

**Figure 1:**
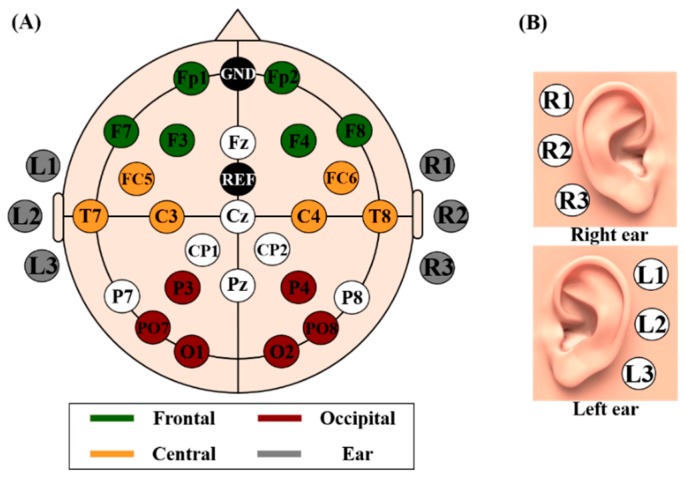
Electrode positions used to record EEG data. (**A**) The brain area is divided into four regions of interests (ROIs) for data analysis (frontal, central, occipital, and ear area). (**B**) The electrode placement for ear-EEG.

EEG data were recorded in a sound-proof room, and subjects were seated in a comfortable armchair in front of a 21-inch monitor about 1 m away. During the experiment, the subjects were asked to refrain from any body movement to minimize physiological artifacts. A binaural audio system (Britz, BR-1000A, Cuve Black2, Paju-si, South Korea) was placed on both sides of the monitor, and it provided the subjects with auditory cues during the experiment. EEG data were recorded using thirty-one electrodes of a multi-channel EEG apparatus (Brain Products, GmbH, Gilching, Germany). Scalp-EEGs were measured using twenty-five electrodes attached to the scalp according to the international 10–20 system (Fp1–2, Fz, F3–4, 7-8, FC5–6, Cz, C3–4, T7–8, CP1–2, Pz, P3–4, 7–8, PO7–8, O1, and O2), while ear-EEG data were measured using six electrodes attached behind the ears (three electrodes for each ear). In order to measure ear-EEG, we first cleaned the skin behind the ears using an alcohol, a double-sided sticker was attached on the skin, a rubber ring holder was mounted on the sticker, and an electrode was inserted into the holder. Same types of electrodes were used to measure both scalp- and ear-EEG. The detailed information of electrode positions for scalp-EEG and ear-EEG is illustrated in Figure 1. Reference and ground electrodes were attached at FCz and Fpz, respectively. The sampling rate was 1000 Hz, and impedance was maintained below 10 kΩ during the entire experiment. The scalp- and ear-EEG were independently re-referenced before the analysis (see Section 2.4 for details).

### 2.3. Experimental Paradigm

Before the main experiment, we measured EEGs when the subjects kept their eyes closed (EC) and eyes opened (EO) for 30 s each, which was repeated six times. The preliminary experiment was conducted to check the feasibility of using ear-EEG by confirming the well-known neurophysiological phenomenon that there is a significant increase in alpha power (8–13 Hz) when the eyes are closed compared to when they are open. 

**Figure 2:**
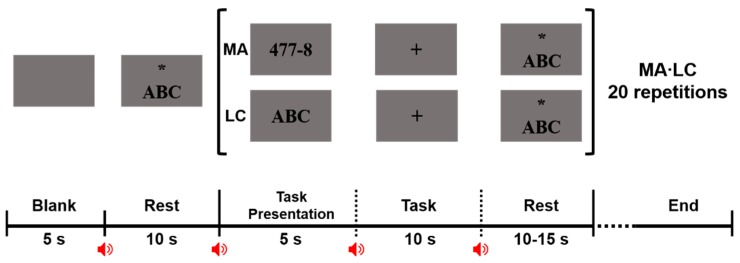
Experimental paradigm of one session used in the main experiment. In the beginning of each session, a rest period of 10 s is performed. The string ‘ABC’ and an asterisk are presented to indicate a rest period and the subject is asked to fix the eyes to the asterisk to minimize ocular movement. After the rest period, either mental arithmetic (MA) or light cognitive (LC) task is randomly performed. For MA, a pair of a three-digit number and a single-digit number between 5 and 9 is randomly presented, and the subject is asked to sequentially subtract the single-digit number from the three-digit number (e.g., 477 − 8) for 10 s. For LC, the string ‘ABC’ is presented, and the subject is asked to internally imagine vocalization of the English alphabet from A to Z with a 1 Hz speed for 10 s. Both MA and LC are performed ten times in each session, and each subject completes five sessions (50 MA and 50 LC in total). A short beep (300 ms) is presented at every screen transition (red speaker icons).

Figure 2 shows the schematic diagram of the main experiment conducted to confirm whether an endogenous BCI paradigm can be realized using only ear-EEG. Two cognitive tasks, MA and LC, were employed in the main experiment. During MA, the subjects were instructed to sequentially subtract a single-digit number (between 5 and 9) from a three-digit number (e.g., 594 − 8) [31]. Fifty pairs of 3-digit and 1-digit numbers were randomly selected and presented to each subject, but the order of the 50 MA tasks was same between the subjects. We asked subjects to perform the consecutive subtraction as quickly as possible during the task period. During LC, they were instructed to mentally imagine vocalization of English letters from A to Z at 1 Hz without vocalization. The LC task was designed to maintain a steady and constant level of light cognitive load, which was introduced because subjects tend to randomly think something that might disturb the low loading state in a conventional resting state [30,31]. All subjects completed five sessions. Each experimental session started with an initial resting state in which a blank was first displayed for 5 s, which was followed by resting state where the string ‘ABC’ with an asterisk were presented. The subjects imagined vocalization of the English alphabet for 10 s while focusing on the asterisk at the center of a monitor screen that was used as a fixation mark to prevent severe ocular movement. A single trial was comprised of a task instruction of 5 s, followed by a task period of 10 s with a black fixation cross, and a variable resting period ranging from 10–15 s. During the task instruction period, either the MA problem or the ‘ABC’ string (LC) was randomly displayed on the screen for 5 s. The task started by presenting a black fixation cross that lasted for 10 s, during which the subjects performed either MA or LC according to an instruction presented on the monitor. The task period was followed by rest. A short beep was presented for 300 ms at every screen transition to provide subjects with explicit information of screen transition. Each session consisted of 10 MA and 10 LC trials, and a short break was given for several minutes between sessions. All subjects performed 50 MA and 50 LC trials (10 trials × 5 sessions).

### 2.4. EEG Data Analysis

EEG data were analyzed using EEGLAB [39,40] and BBCI toolbox [39,40] based on MATLAB (MathWorks, Natick, MA, USA). The EEG data were first bandpass-filtered between 1 and 50 Hz using a zero-phase fourth-order Butterworth filter and then down-sampled to 200 Hz in order to avoid introducing unwanted artifacts after downsampling. To compare neural characteristics and classification performance of different brain areas, we divided the brain area into four regions of interests (ROIs): frontal, central, occipital, and ear area (see Figure 1 for more detail about the four ROIs). For fair comparison, we selected six electrodes for each ROI, as follows: frontal (Fp1–2, F3–4, 7–8); central (FC5–6, C3–4, T7–8); occipital (P3–4, PO7–8, O1–2); and ear area (R1–3, L1–3). All ROIs were re-referenced to remove the impact of the original reference electrode (FCz). Three scalp ROIs were independently re-referenced using a common average reference (CAR) method with six electrodes each [41] while ear-area was re-referenced using a modified CAR in which the mean of three electrodes attached on an opposite ear area was used as a reference signal [16]. Trials contaminated by eye blinks and body movements were removed based on a peak-to-peak amplitude thresholding method [42]. Two samples showing the lowest and highest amplitudes were first found for each component caused by eye blink and the difference between them was calculated. If the difference was higher than a certain threshold at least once in the trial, a corresponding trial was removed. A threshold differed between subjects because a baseline amplitude varied from one subject to others (126.67 ± 22.95 μV for MA and LC; 196 ± 51.34 for EO/EC). The subjects blinked the eyes relatively strongly in the preliminary experiment as compared to in the main experiment, in particular, during the transition between EC and EO to get vision back. Note that EC was first performed, followed by EO. Thus, the threshold for EO/EC was set to be higher than that of MA and LC. The numbers of rejected trials for MA, LC, and EO/EC were 4 ± 1.66, 3.66 ± 1.06, 1.27 ± 0.77 in average, respectively. 

For the EEG data measured in the preliminary experiment with EC and EO, time-frequency analysis was performed using a short-time Fourier transformation (window size: 1 s, 50% overlap). Spectral powers in a frequency band from 5–15 Hz, containing the α-band (8–13 Hz), were used to investigate alpha power changes between the EC and EO conditions. Relative changes of mean alpha power from EO to EC (signal-to-noise ratio: SNR) were estimated in decibels for the four ROIs (frontal, central, occipital, and ear area). The SNR is simply given as:

(1) $$\mathrm{SNR}=10\times \log_{10}\left(\frac{alpha_{EC}}{alpha_{EO}}\right)$$

For the EEG data measured during MA and LC in the main experiment, ERD/ERS analysis was first performed [43], for which epochs from −2–10 s based on the task onset of MA and LC were extracted. Baseline correction was performed by subtracting the mean value of the EEG data recorded between −2–0 s from each data point. ERD/ERS patterns induced during MA and LC were calculated for each channel, and averaged over the six channels contained in each ROI. Classification of MA and LC was performed for each ROI. For feature extraction, a multi-band common spatial pattern (CSP) was applied to the epochs of MA and LC [44,45], where five frequency bands were used: δ-band (1–3 Hz), θ-band (4–7 Hz), α-band (8–13 Hz), β-band (14–29 Hz), and γ-band (30–50 Hz). The log-variances of the two first and last CSP components were calculated in each band as features for classification. Note that the multi-band CSP was applied to each ROI individually. To estimate classification accuracy, ten-fold cross-validation was performed ten times using shrinkage linear discriminant analysis (sLDA) [46,47]. All statistics were carried out using a Friedman test, and a Wilcoxon signed-rank sum test was used for post-hoc analysis with Bonferroni correction (i.e., *p* = 0.05/number of post-hoc tests).

## 3. Results

### 3.1. Alpha Power Changes during EC and EO

**Figure 3:**
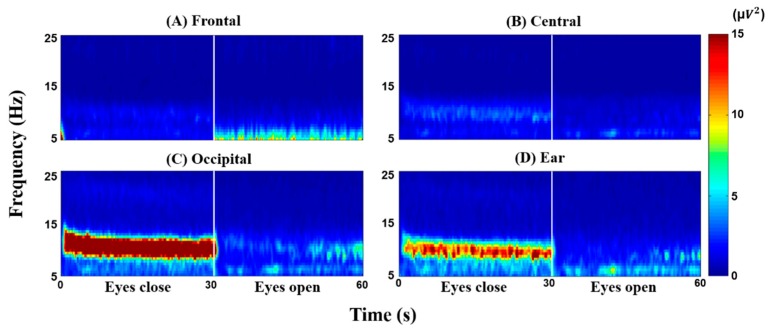
Grand average time-frequency maps with eyes closed (EC) and eyes opened (EO) for (**A**) frontal, (**B**) central, (**C**) occipital, and (**D**) ear area. The color scale was chosen to fit the range for (**D**) ear area.

Figure 3 illustrates grand-average time-frequency maps of scalp- and ear-EEG in the frequency band from 5–15 Hz. As is well documented, a more significant increase in alpha power—around 10 Hz—is observed in the occipital area during EC than during EO (Figure 3C). Interestingly, a similar pattern is also shown for ear-EEG (Figure 3D), which can be explained by the fact that ear-EEG is measured close to the occipital lobe that mainly shows an alpha power increase during EC. Frontal and central areas also show an alpha power increase during EC, but which is not as strong as occipital and ear areas. The SNRs of the frontal, central, occipital, and ear area (EC/EO) are 0.74 ± 0.40, 1.55 ± 0.54, 4.20 ± 3.38, and 2.48 ± 1.32, respectively. As expected, the mean SNR of the occipital area is the highest and is significantly higher than the other ROIs. The SNR of the ear area is also statistically higher than both frontal and central areas (Wilcoxson signed rank sum test; occipital > ear > central > frontal, corrected *p* < 0.05).

### 3.2. ERD/ERS Pattern Maps during MA and LC

**Figure 4:**
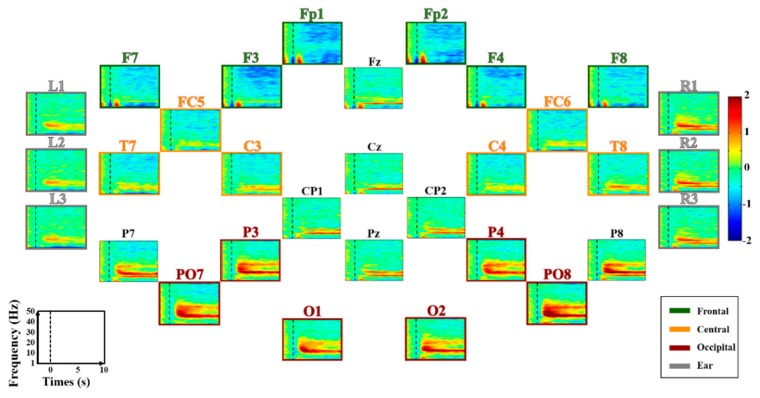
Grand average ERD/ERS maps of all electrodes during MA. The four regions of interest (ROIs), frontal, central, occipital, and ear area, are denoted by four different colored lines and titles for each map (green, orange, red, and gray), respectively. The x- and y-axis of each map indicate the task time from −2–10 s based on task onset (t = 0 s), and the frequency band ranging from 1 to 50 Hz, respectively. ERD and ERS are presented in blue and red, respectively. Note that scalp- and ear-EEG are independently re-referenced using a CAR and a modified CAR, respectively.

**Figure 5:**
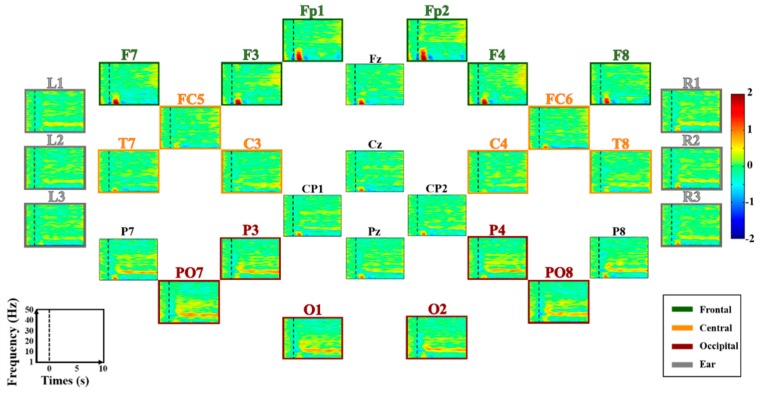
Grand average ERD/ERS maps of all electrodes during LC. The four regions of interest (ROIs), frontal, central, occipital, and ear area, are denoted by four different colored lines and titles for each map (green, orange, red, and gray), respectively. The x- and y-axis of each map indicate the task time from −2–10 s based on task onset (t = 0 s), and the frequency band ranging from 1 to 50 Hz, respectively. ERD and ERS are presented in blue and red, respectively. Note that scalp- and ear-EEG are independently re-referenced using a CAR and a modified CAR, respectively.

**Figure 6:**
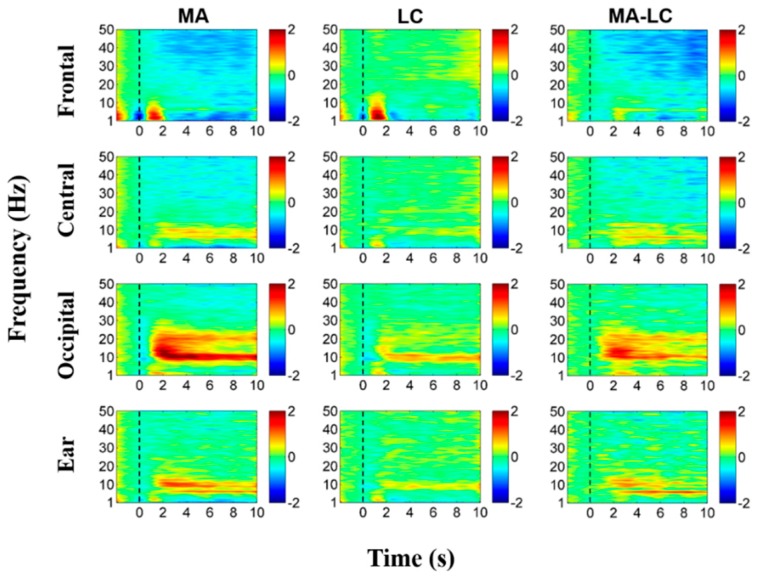
Grand average ERD/ERS maps of each ROI during MA and LC, and their differences (MA-LC). The ERD/ERS maps of ear area (denoted by ‘Ear’) are obtained by averaging the six electrodes attached behind both ears (three electrode for each side). The x- and y-axis of each map indicate the task time from −2–10 s based on task onset (t = 0 s), and the frequency band ranging from 1 to 50 Hz, respectively. ERD and ERS are presented in blue and red, respectively.

Figure 4 shows grand average ERD/ERS maps of all electrodes during MA. Prominent ERS is observed in the α-band around 10 Hz at most electrodes, while a wide ERD is observed in the β- and γ-bands [32,33]. ERS is stronger in occipital and ear areas than fronto-central areas; an opposite trend is observed for ERD. Grand average ERD/ERS patterns of all electrodes during LC are shown in Figure 5. There are no distinct ERD/ERS patterns compared to those induced during MA. However, natural α-oscillation is visibly observed around the parieto-occipital areas and their adjacent ear areas, but it is not as strong as the alpha ERS induced during MA. Figure 6 presents grand average ERD/ERS pattern maps during MA and LC along with the difference between MA and LC (denoted by ‘MA-LC’) for the four ROIs. 

The ERD/ERS patterns shown in Figure 4 and Figure 5 can be similarly observed for each ROI, and the difference between MA and LC is clearly seen (see ‘MA-LC’), leading to reasonable classification between the two conditions. Interestingly, ERD/ERS patterns for the ear area are very similar to those for the central and occipital areas adjacent to the ear area, demonstrating the feasibility of using ear-EEG to develop cognitive-task-based endogenous BCIs.

### 3.3. Classification Performance

**Figure 7:**
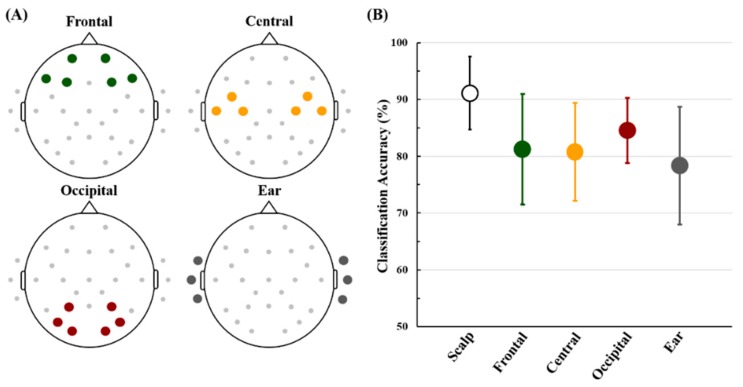
(**A**) Electrode positions used to create each ROI, and (**B**) the mean classification accuracies of the four ROIs with that obtained using all electrodes (‘Scalp’), excluding the six ear electrodes. Each ROI was individually re-referenced, where a CAR was used for the scalp ROIs (‘Scalp’, ‘Frontal’, ‘Central’, and ‘Occipital’) while the mean of three electrodes attached on an opposite ear area was used as a reference signal for ear ROI (‘Ear’). Error bars indicate standard deviations of the estimated classification accuracies of each ROI. There is no significant difference between the four ROIs (Friedman test; *p* = 0.63).

Figure 7 shows the electrode positions of each ROI and their mean classification accuracies. The classification accuracy attained using all electrodes re-referenced using CAR, excluding the six ear electrodes, is also presented as a reference accuracy. The mean accuracies of the frontal, central, occipital, and ear areas are 81.26 ± 9.72, 80.78 ± 8.60, 84.55 ± 5.75 and 78.36 ± 10.36%, respectively (all electrodes: 91.80 ± 5.60%). The classification accuracy of the occipital area is higher than those of the other ROIs, but there is no statistically significant difference between the four ROIs (Friedman test; *p* = 0.63).

## 4. Discussion

In recent years, ear-EEG has been introduced to develop a more practical BCI system as an alternative to the classical scalp-EEG; its feasibility has been demonstrated in many previous studies [15,16,17,23,25,26,27,48,49,50,51,52,53]. Most of previous studies have used exogenous BCI paradigms that take advantage of brain activity evoked by external stimuli, such as ASSR [16,18,23,48,54,55,56,57], SSVEP [23,51,55,57], and ERP [15,17,23,25,26,27,48,49,58], while few studies based on ear-EEG have developed endogenous BCI systems based on self-modulated brain activity. Development of an endogenous BCI system is important because continuous external visual or auditory stimulation can readily cause fatigue [29,59,60], and the performance of these BCI systems is significantly degraded when the user has deficits in visual or auditory function [59,61]. The goal of this study was to investigate whether ear-EEG can be used to develop an endogenous BCI system with reliable performance. To this end, we conducted preliminary and main experiments to check the feasibility of using an ear-EEG based on the EC/EO task and an endogenous BCI paradigm (MA vs. LC), respectively.

In the preliminary experiment, we compared changes in alpha activity induced during EC and EO for each ROI, and we confirmed that the SNR of ear-EEG was comparable even though it was smaller than that of the occipital area, which showed the highest increase of alpha activity during EC. This result demonstrated the feasibility of using ear-EEG as compared to traditional scalp-EEG, and is in line with previous reports [17,25,57]. Even though the previous studies investigated alpha power changes induced during EC and EO, they only checked them using ear-EEG without direct comparison between ear-EEG and scalp-EEG. So, it should be noted that this study is first to quantitatively compare the SNR of ear-EEG with those of other brain areas in terms of alpha activity increase related to EC. The comparison presented in this study could provide more detailed information about the degree of utilization of ear-EEG as compared to scalp-EEG in developing EEG applications based on alpha activity, such as brain authentication based on the resting state [62], attention monitoring [63,64], and sleep detection [65].

In the main experiment, we used a MA task that is one of the widely employed cognitive tasks for developing an endogenous BCI system [31,33,66,67,68,69]. A significant alpha ERS and a wide-band ERD in the β- and γ-bands were observed for most electrodes during MA, which is consistent with previous studies [32,33]. Most importantly, similar ERD/ERS patterns were also shown for ear-EEG during MA. Also, the ERD/ERS pattern maps of the ear area were more similar to those of centro-occipital areas than those of frontal areas, which was also indirectly proved in a previous study showing that centro-occipital EEGs can be more predicted using ear-EEG than frontal EEG [70]. As documented in related studies [33,71], the strongest alpha ERS was observed during MA on occipital areas that are most sensitive to cognitive tasks [33], thereby showing the highest classification accuracy between MA and LC (84.55 ± 5.75%). Importantly, the classification performance of the ear area was comparable to the other ROIs from the statistical point of view (Friedman test; *p* = 0.63). In addition, the mean classification accuracy of ear-EEG exceeded the marginal accuracy of 70% that determines whether a binary BCI system can be practically used [72]. The ERD/ERS and classification results could demonstrate that ear-EEG can be used to develop an endogenous BCI system with acceptable performance.

In this study, we used MA and LC (mental vocalization task) tasks to confirm the feasibility of ear-EEG for the development of an endogenous BCI system. So far, various mental tasks have been used to develop endogenous BCI systems, such as motor imagery [34,73], mental rotation [74], spatial navigation [75], mental vocalization [76,77], and word association [78]. Thus, further studies for other mental tasks should be performed to more generally address the feasibility of ear-EEG for developing an endogenous BCI system, which could also provide the information about proper combinations of mental tasks for implementing ear-EEG-based BCIs. On the other hand, we used one of the language tasks as a LC task because it was confirmed that the LC task used in this study can maintain constant level of low cognitive loading [31]. However, considering that a mental subtraction task was used as a main task in this study in order to actively induce self-modulated EEGs, an easy subtraction task (e.g., 100 − 1) might be a more proper selection as a LC task instead of the language task for the sake of task unity. Investigating the difference between the language task used in this study and an easy subtraction task will be considered in terms of neural characteristics and task performance in future studies.

A multi-band CSP and sLDA were used for feature extraction and classification, respectively, in this study. However, because there is no guarantee that sLDA is always most suitable for classification of given EEG data, another classifier can be considered. For example, random forest, *k*-nearest neighbor, and Gaussian mixture model showed better classification performance than LDA for sleep EEGs [79]. Because a most suitable classifier highly depends on the characteristics of given dataset, trial and error with different classifiers should be undergone to find a better classifier [80]. In this study, sLDA was a better choice than other two classifiers tested, random forest and support vector machine, in terms of classification accuracy (not shown here in detail).

As mentioned above, most ear-EEG studies have used external auditor/visual stimuli and have demonstrated that ear-EEG can reliably capture evoked brain potentials, such as SSVEP [23,51,55,57], ASSR [16,18,23,48,54,55,56,57], and ERP [15,17,23,25,26,27,48,49,58]. In this study, we demonstrated that ear-EEG can also measure self-modulated brain activity (Figure 6). Thus, it can be thought that spontaneous brain activity generated without the repetitive use of external stimuli and the execution of mental tasks might be captured using ear-EEG. Thus, investigation of spontaneous brain activity measured around the ears would be an interesting future research topic to expand the application areas of ear-EEG, such as emotion recognition and epileptic seizure detection.
